# 球形氨基功能化共价有机骨架化合物的制备及对全氟化合物的吸附性能考察

**DOI:** 10.3724/SP.J.1123.2022.11013

**Published:** 2023-06-08

**Authors:** Junbin YE, Jiawei LIU, Anqi CUI, Xiaoyi WU, Hui SUN

**Affiliations:** 广州大学环境科学与工程学院, 广东 广州 510006; School of Environmental Science & Engineering, Guangzhou University, Guangzhou 510006, China

**Keywords:** 共价有机骨架, 点击反应, 全氟化合物, covalent organic frameworks (COFs), click reactions, perfluorinated compounds (PFCs)

## Abstract

全氟化合物(PFCs)是一类环境持久性污染物,对生态系统和人类健康造成了极大威胁。一般环境水体中PFCs含量很低,高性能萃取材料的制备和应用一直是近年来的研究热点。为了有效富集水中的PFCs,本研究通过“两步法”制备出氨基功能化的球形共价有机骨架(COF)材料。首先利用1,4-二醛基-2,5-二乙烯基苯及1,3,5-三(4-氨苯基)苯为构筑基元,通过调控反应条件在室温下合成出粒径均匀的球形乙烯基COF(Vinyl COF)材料;然后以4-氨基苯硫酚为氨基功能化单体,通过巯基-烯基点击反应引入功能化侧基,合成出硫醚桥连芳香胺功能化球形COF材料(COF-NH_2_)。该材料具有丰富的氨基,能够与PFCs碳链上的氟基以及羧基发生多重氢键和静电作用,因此可以有效吸附PFCs。本论文探究了COF-NH_2_的吸附动力学、等温吸附模型、再生性能以及不同酸度条件下的吸附性能。COF-NH_2_微球直径约500 nm,具有较好的热稳定性,可以在较宽的pH范围内有效吸附PFCs;重复再生5次后,吸附效果几乎无影响。该材料在实际环境水体中具有良好的吸附效能,对自来水和珠江水样中5种PFCs(全氟丁酸、全氟戊酸、全氟己酸、全氟辛酸及全氟壬酸)的萃取效率为91.76%~98.59%。该球形COF材料尺寸均匀,热稳定性好,具有较好的吸附性能而且容易再生,可望用作固相萃取填料或者液相色谱柱固定相用于PFCs的高效富集分离和检测,具有较好的应用前景。

## Abstract (en)

Perfluorinated compounds (PFCs) are widely used in textiles, fire protection, metal electroplating, and semiconductor production owing to their hydrophobic and oil-repellent characteristics. However, they are also persistent organic pollutants. The uncontrolled discharge of PFCs into the environment has led to serious global pollution. PFCs pose severe reproductive, neural, immune, and other threats to human health by accumulating through the food chain. Thus, the development and application of high-performance extraction materials has become a research hotspot in efforts to achieve the accurate detection of trace PFCs in environmental waters. Most traditional PFC adsorbents present a number of disadvantages, such as low adsorption selectivity, slow diffusion, and poor reusability. Covalent organic frameworks (COFs) are crystalline polymers with ordered porous structures, large specific surface areas, and high chemical and thermal stability. These frameworks can easily be functionalized for the desired purpose. In this paper, spherical amino-functionalized COFs (denoted COF-NH_2_) were fabricated via a two-step method to effectively enrich/remove PFCs from water. First, vinyl covalent organic framework (Vinyl COF) was synthesized at room temperature using 1,4-diradical-2,5-divinylbenzene (Dva) and 1,3,5-tris(4-aminophenyl)benzene (Tab) as building blocks. Then, thioether-bridged aromatic amine-functionalized spherical COF-NH_2_ was synthesized through a thiol-alkenyl click reaction using 4-aminothiophenol as the functional monomer. COF-NH_2_ showed good dispersion in water owing to its abundant amino groups, forming multiple hydrogen bonds with the F atoms of PFCs. The synergistic hydrophobic interactions between the organic skeleton of the COF and alkyl carbon chains of the PFCs led to enhanced adsorption efficiency. The produced Vinyl COF and COF-NH_2_ were characterized by Fourier transform infrared spectroscopy (FT-IR), field-emission scanning electron microscopy (SEM), powder X-ray diffraction (PXRD), thermogravimetric analysis (TGA), and Brunner-Emmet-Teller (BET) measurements. The results confirmed that spherical COF-NH_2_ materials with a homogeneous size distribution were successfully fabricated. The obtained COF-NH_2_ microspheres had a diameter of approximately 500 nm and exhibited high thermal stability as well as a large specific surface area and pore volume. The adsorption kinetics, isotherm adsorption models, pH effects, and regeneration properties of COF-NH_2_ were also investigated, and the results indicated that the adsorption of PFCs by COF-NH_2_ conformed to the pseudo-second-order kinetic and Langmuir isotherm adsorption models. The obtained COF-NH_2_ microspheres can be applied over a wide pH range, and the best adsorption effect was achieved in neutral and alkaline environments. After five cycles of regeneration and reuse, the COF-NH_2_ microspheres retained their good adsorption efficiency for PFCs. The adsorption mechanism was mainly attributed to the synergistic effect of hydrogen bonding and hydrophobic interactions between COF-NH_2_ and the PFCs. The extraction efficiencies of the microspheres toward five PFCs (perfluorobutyric acid, perfluorovaleric acid, perfluorohexanoic acid, perfluorooctanoic acid, and perfluorononanoic acid) in tap and Pearl River water samples were between 91.76% and 98.59%, with relative standard deviations (RSDs) (*n*=3) varying from 0.82% to 3.8%; these findings indicate that the obtained COF-NH_2_ is promising for the extraction of PFCs from complex water samples. Given their uniform size distribution, high thermal stability, good adsorption performance, and reusability, the novel spherical COF-NH_2_ materials developed in this study may be used as solid-phase extraction materials or filled into liquid chromatographic columns for the enrichment, separation, and detection of PFCs in complex samples.

全氟化合物(perfluorinated compounds, PFCs)是一种疏水、疏油、耐高温、化学稳定性好并能够改变水的表面张力的化合物,它们被广泛应用于纺织、消防、金属电镀以及半导体生产等行业中^[1]^。PFCs主要通过工业排放以及日常生活用品的释放而进入环境,并能通过饮食摄入、皮肤接触、家居灰尘、空气吸入、胎盘转移等途径在人体内积聚^[2]^。国内外人员的调查研究发现,PFCs具有生殖、神经、免疫等多种毒性,并且能够通过食物链进行累积,进而呈现出全球生态污染的趋势^[3]^。由于PFCs的长期广泛使用及在环境中的生态持久性,近年来这类化合物已经陆续在全世界范围内的各类环境介质及生物体内检出。PFCs中含有强极性的碳氟键,因此具有很高的稳定性,难以通过光解、水解、氧化、微生物降解、生物代谢等过程消除。除了全氟辛酸(perfluorooctanoic acid, PFOA)及全氟辛烷磺酸基化合物(perfluorooctane sulfonate, PFOS)之外,其他各种PFCs在环境中也日益增多,对生态系统和人类健康造成了极大威胁^[4]^。

一般环境水体中PFCs含量很低,通常需要先进行富集浓缩,然后进行检测。固相萃取(SPE)是富集或去除废水中PFCs等许多污染物的有效和经济的方法。吸附材料的性能直接影响着萃取行为,故新型高性能萃取材料的制备和应用一直是近年来的研究热点^[5]^。活性炭^[6]^、离子交换树脂^[7]^等传统的吸附剂存在吸附选择性差、孔隙扩散缓慢、洗脱条件较苛刻、难以重复利用、吸附时间长等缺点,在实际应用中对PFCs等目标化合物的萃取能力往往有限。为了克服以上问题,各种新型PFCs萃取材料如碳纳米管^[8]^、分子印迹聚合物^[9]^、金属有机框架材料(metal organic framework, MOF)^[10⇓⇓⇓-14]^和共价有机骨架(covalent organic framework, COF)材料^[15,16]^等被设计合成出来。尤其是MOF和COF材料具有牢固的网络结构,内部化学环境可调,骨架内通道相连,可再生,易于功能化,传质速度快,给PFCs的吸附处理带来了光明的前景。如Li等^[14]^发现NU-1000锆基MOF对全氟羧酸(perfluorinated carboxylic acid, PFCA)的吸附能力为201~604 mg/g,且具有超快的吸附动力学,其平衡时间小于1 min。但是在酸性溶液中MOF金属中心与有机配体之间的配位键会发生解离,导致其骨架不稳定,引起坍塌等现象,因此其应用受到一定限制。与MOF不同,COF是由C、H、O、N、B、Si等第一、二周期的轻元素通过可逆的强共价键连接而成的纯有机晶态多孔材料。COF材料具有极低的骨架密度,较高的比表面积,优异的酸碱及热稳定性,而且具有丰富的构筑单体、多样的桥连形式,可以根据需求在骨架上引入特定的预置基团,使其具备功能上的可调控性,可根据目标物制备出高选择性的COF材料^[15,16]^。因此COF在各个领域,包括催化、光电、气体储存、吸附分离水中有害物以及药物控释等方面,展现出了潜在优势^[17,18]^。为了有效去除水环境中的PFCs或其他污染物,人们相继发展了单体功能化、桥连基团转换等策略,制备出了具有特定功能结构的新型COF^[19⇓⇓-22]^。研究表明,静电、氢键和疏水作用是控制PFCs在COF吸附剂上吸附的主要作用力^[23,24]^。

然而COF材料一般由芳香环通过不同化学键桥连得到,多数COF是高度疏水的,在水基样品中容易团聚,影响对目标底物的萃取效果。而且不均匀形状的COF直接作为固相萃取填料容易导致柱压过高、材料渗漏等问题,从而限制了其应用。为满足不同环境样品前处理技术的要求,研究者们通常将COF与树脂、二氧化硅、四氧化三铁等构成复合材料使用,但该过程复杂,同样限制了COF在样品预处理领域的应用^[24]^。考虑到球形COF材料与一般COF材料相比,具有理化性能稳定、尺寸均匀、传质快速、易于进行表面修饰等优点,而且便于填充到液相色谱柱或固相萃取柱中,或者用做传感材料增加对PFCs的富集分离和检测应用范围,因此本研究拟制备出氨基功能化的球形COF材料(COF-NH_2_);该材料表面富含氨基,一方面可以提高COF材料在水介质中的分散性,另一方面可以与PFCs碳链上的氟基或羧基产生多重氢键或静电作用增加对PFCs的吸附能力。

本研究首先利用1,4-二醛基-2,5-二乙烯基苯(Dva)以及1,3,5-三(4-氨苯基)苯(Tab)为构筑基元,调控反应条件,室温合成出单分散性良好的球形乙烯基COF(Vinyl COF)材料;然后进一步以4-氨基苯硫酚为功能单体,利用巯基-烯基点击反应在乙烯基COF表面上接枝亚胺基团,合成出硫醚桥连芳香胺功能化球形COF材料(COF-NH_2_),并探究了该材料的吸附动力学、热力学、再生性能以及在实际样品中对PFCs的吸附性能。

## 1 实验部分

### 1.1 仪器与试剂

JSM-7001F热场发射扫描电镜(SEM,北京捷欧路科贸有限公司); HWY-100B恒温振荡摇床(广州科桥实验技术设备有限公司); DF-101S集热式恒温加热磁力搅拌器(巩义市予华仪器有限责任公司); SB 5200 DTS双频超声波清洗机(宁波新芝生物科技股份有限公司); BSA224S电子天平(赛多利斯科学仪器有限公司); DZX-6050B恒温干燥箱(上海福码实验设备有限公司); VERTEX70傅里叶变换红外光谱仪(FT-IR,德国Bruker公司); Rigaku9000粉末X射线衍射仪(PXRD,日本理学公司); H1650-W高速离心机(湖南湘仪仪器有限公司); QL-866旋涡混合器(海门市其林贝尔仪器制造有限公司); Agilent 1260-6460液相色谱-三重四极杆质谱联用仪(HP-MS,美国安捷伦科技公司)。

Dva(纯度97%)与Tab(纯度98%)购于上海腾骞生物科技有限公司;三氟甲苯(纯度99%)、4-氨基苯硫酚(纯度97%)、全氟丁酸(perfluorobutyric acid, PFBA,纯度98%)、全氟戊酸(perfluorovaleric acid, PFPeA,纯度97%)、全氟己酸(perfluorohexanoic acid, PFHxA,纯度98%)、全氟辛酸(纯度98%)、全氟壬酸(perfluorononanoic acid, PFNA,纯度97%)及乙酸铵(色谱纯)购于上海麦克林生化科技有限公司;四氢呋喃、无水乙醇、冰乙酸、乙腈及甲醇均为分析纯,购于天津市致远化学试剂有限公司;偶氮二异丁腈(2,2'-azobis(2-methylpropionitrile), AIBN)购于天津市大茂化学试剂厂。

### 1.2 COF材料的合成

#### 1.2.1 乙烯基COF的合成

参考Ma等^[25]^的方法,室温合成出具有良好结晶性的球形乙烯基COF材料。在10 mL离心管中依次加入Dva(14.3 mg, 0.04 mmol)、Tab(11.5 mg, 0.06 mmol)和5 mL乙腈,超声1~2 min使其溶解。在管中再加入0.4 mL 6 mol/L乙酸,使用旋涡混合器混合10 s后,置于安全位置静置3 d得到黄色沉淀。将产物用四氢呋喃和乙醇各清洗3次,置于60 ℃烘箱中真空干燥得到黄色产物。乙烯基COF的合成路线见图1。

**图1:**
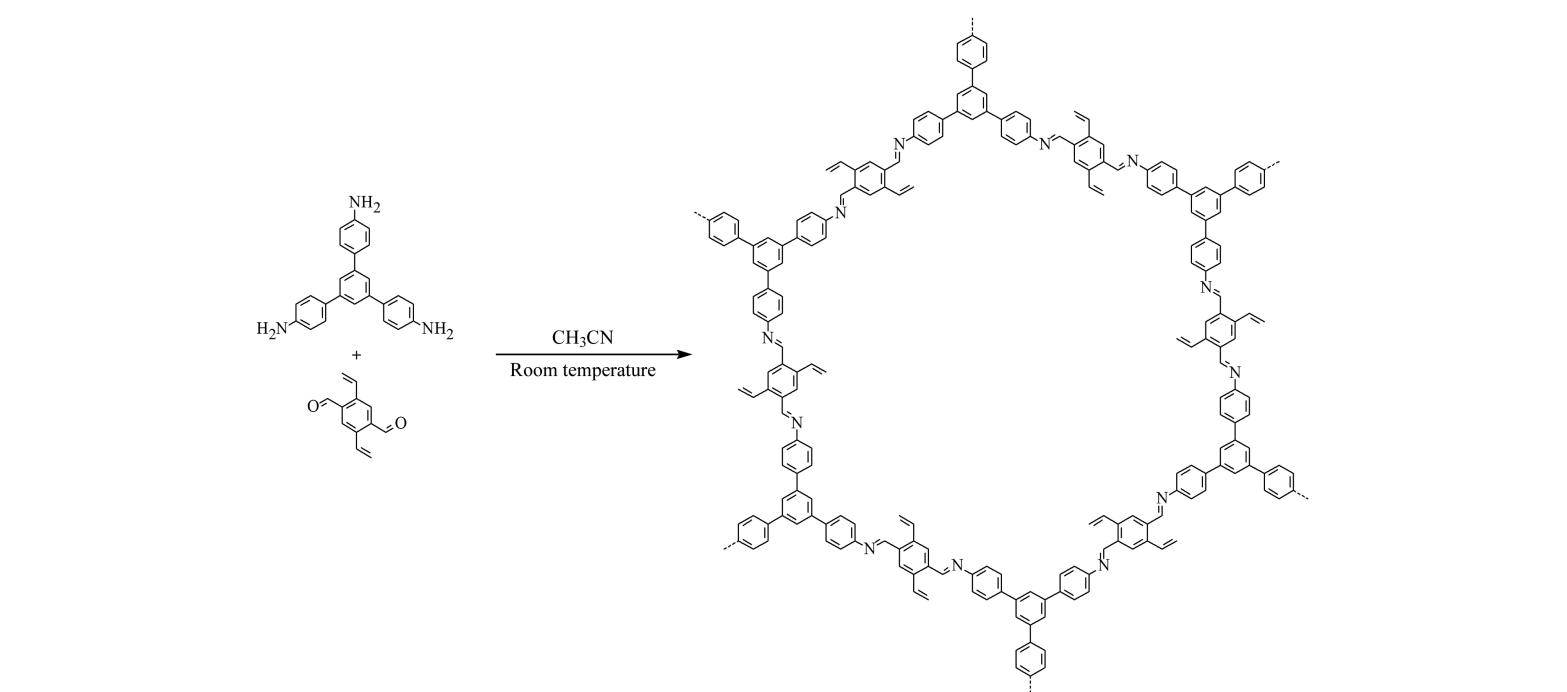
乙烯基COF的合成路线

#### 1.2.2 COF-NH_2_的合成

参考Ji等^[26]^的方法,在乙烯基COF表面上接枝亚胺基团。在圆底烧瓶中加入制备好的乙烯基COF(50 mg)、4-氨基苯硫酚(250 mg)、AIBN(5 mg)和三氟甲苯4 mL, N_2_条件下90 ℃加热6 h。在恢复室温后,用四氢呋喃清洗若干次,置于60 ℃烘箱中真空干燥得到棕黄色产物。COF-NH_2_的合成路线如图2所示。

**图2:**
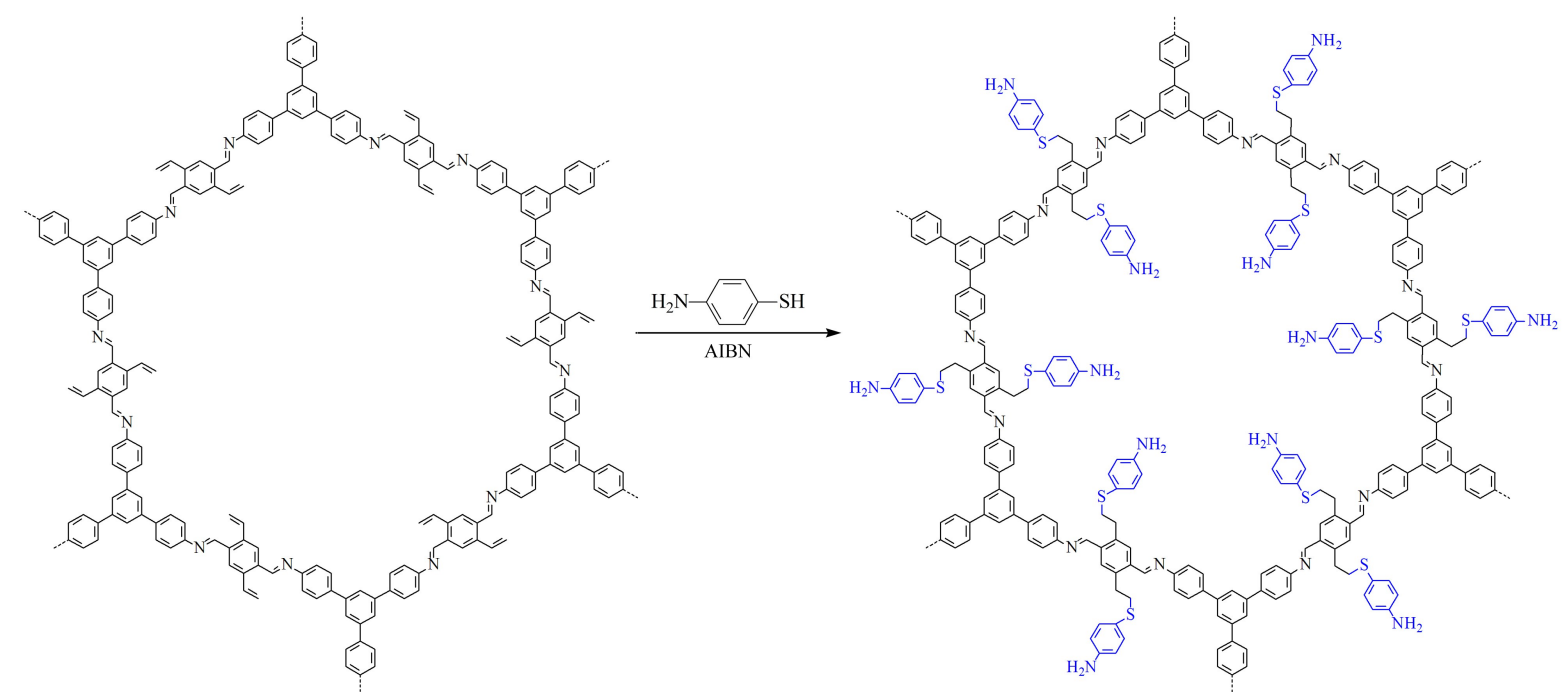
COF-NH_2_的合成路线

### 1.3 HPLC-MS/MS条件

HPLC条件 Waters Atlantis dC18色谱柱(150 mm×2.1 mm, 5.0 μm),流速0.3 mL/min,进样体积10 μL,流动相A为20 mmol/L乙酸铵水溶液,流动相B为甲醇。梯度洗脱程序:0~1 min,流动相A为60%; 1~25 min,流动相A由60%降至10%,保持7 min;32.1 min时,流动相A升回至60%,并保持4.9 min。37 min内完成分离检测。

质谱条件 电喷雾电离(ESI),负离子模式;毛细管电压30 kV;锥孔电压55.0 V;萃取电压3.0 V;透镜电压1.5 V;锥孔气流速50 L/h;雾化气流速800 L/h;离子源温度120 ℃;雾化气温度350 ℃;碰撞能量10 eV;多反应监测(MRM)模式;其他质谱参数见表1。

**表1:** 目标化合物的MRM参数

Analyte	Parent ion (m/z)	Fragment ion (m/z)	Collision energy/eV	Cone voltage/V
PFBA	213.0	169.0	14	10
PFPeA	262.0	219.0	14	10
PFHxA	363.0	318.8	14	10
PFOA	413.0	368.8	14	10
PFNA	463.0	419.0	163	10

PFBA: perfluorobutyric acid; PFPeA: perfluorovaleric acid;PFHxA: perfluorohexanoic acid; PFOA: perfluorooctanoic acid; PFNA: perfluorononanoic acid.

### 1.4 材料的吸附性能

#### 1.4.1 吸附动力学实验

称取5 mg COF-NH_2_于5 mL离心管中,在管中加入5 mL 100 mg/L的PFOA溶液,室温振荡吸附一段时间(0~8 h),分别在0、1、5、15、30、60、120、240、360、480 min时间点取上清液0.5 mL,用0.22 μm微孔滤膜过滤后以HPLC-MS/MS测试滤液中PFOA的残留浓度。根据式(1)计算COF-NH_2_在不同时间点对PFOA的吸附量。


(1) $Q_{t}=\frac{(C_{i}-C_{t})v}{m}$

式中,*Q_t_*(mg/g)为时间*t*时的吸附量,*C*_i_(mg/L)为初始质量浓度,*C_t_*(mg/L)为*t*时的质量浓度,*v*为吸附溶液体积(L), *m*为COF-NH_2_的质量(g)。

#### 1.4.2 等温吸附实验

以甲醇为溶剂制备质量浓度为1000 mg/L的PFOA标准储备液,并在4 ℃下避光储存。使用前用去离子水适当稀释,得到100、125、150、175及200 mg/L的标准溶液。

分别称取2 mg COF-NH_2_于一系列2 mL离心管中,再分别加入2 mL不同质量浓度的PFOA标准溶液,室温振荡吸附6 h。上清液用0.22 μm微孔滤膜过滤,以HPLC-MS/MS测试滤液中PFOA的残留浓度。根据式(2)计算COF-NH_2_对PFOA的饱和吸附量。


(2) $Q_{e}=\frac{(C_{i}-C_{e})v}{m}$

式中,*Q*_e_(mg/g)为饱和吸附量,*C*_e_(mg/L)为平衡质量浓度,*v*为吸附溶液体积(L)。

#### 1.4.3 最佳pH的确定

分别移取2 mL pH=4、6、7、8、10的100 mg/L PFOA溶液于离心管中,各投加2 mg COF-NH_2_,于25 ℃恒温振荡6 h后,上清液用0.22 μm微孔滤膜过滤,以HPLC-MS/MS测试滤液中PFOA的残留浓度。

#### 1.4.4 不同种类PFCs吸附实验

首先以甲醇为溶剂,分别配制出质量浓度为1000 mg/L的PFBA、PFPeA、PFHxA、PFOA、PFNA单一标准储备液。然后分别用PBS缓冲溶液(0.01 mol/L, pH 7) 稀释,配制出10 mg/L的PFBA、PFPeA、PFHxA、PFOA和PFNA单一标准溶液。向1 mL上述5种PFCs标准溶液中分别加入1.0 mg COF-NH_2_, 25 ℃下振荡6 h,达到吸附平衡。吸附完成后,取上清液用0.22 μm微孔滤膜过滤后以HPLC-MS/MS测试滤液中每种全氟化合物的残留浓度。根据式(1)计算COF材料对不同全氟化合物的吸附量。

#### 1.4.5 COF材料再生实验

收集吸附过全氟化合物的COF-NH_2_材料,加入2 mL甲醇,使用旋涡混合器混合15 min,过滤后用去离子水洗涤3次,60 ℃下真空干燥6 h,备用。将解吸后的COF-NH_2_用于上述吸附过程,测试再生能力。

### 1.5 实际应用

本实验选取珠江水及自来水样品进行吸附研究。其中,珠江水收集于广州大学附近的珠江口(经度113°,纬度23°),珠江水的pH为7.8,电导率为818 μS/cm;自来水采自广州大学实验室用水系统,pH为7.7,电导率为139 μS/cm。上述水样经过0.22 μm的水相滤膜过滤后使用。分别将1 mg吸附剂投入到1 mL水样中,振荡吸附6 h。上清液用0.22 μm微孔滤膜过滤,以HPLC-MS/MS测试滤液中PFBA、PFPeA、PFHxA、PFOA、PFNA的浓度。

## 2 结果与讨论

### 2.1 COF材料的表征

通过SEM观察到,乙烯基COF为单分散性良好的、直径约500 nm的球形COF材料(图3a)。PXRD表征结果证明该材料具有良好的晶态(图4)。对比乙烯基COF和配体的红外光谱(图5),可以看出,在乙烯基COF材料的红外光谱图中,配体Dva在1690 cm^-1^处的C=O特征峰和Tab在大于3200 cm^-1^处的N-H特征峰已经消失,而在1625 cm^-1^处出现了C=N特征峰,这可以证明两种配体发生缩合反应得到了C=N键连接的乙烯基COF材料。

**图3:**
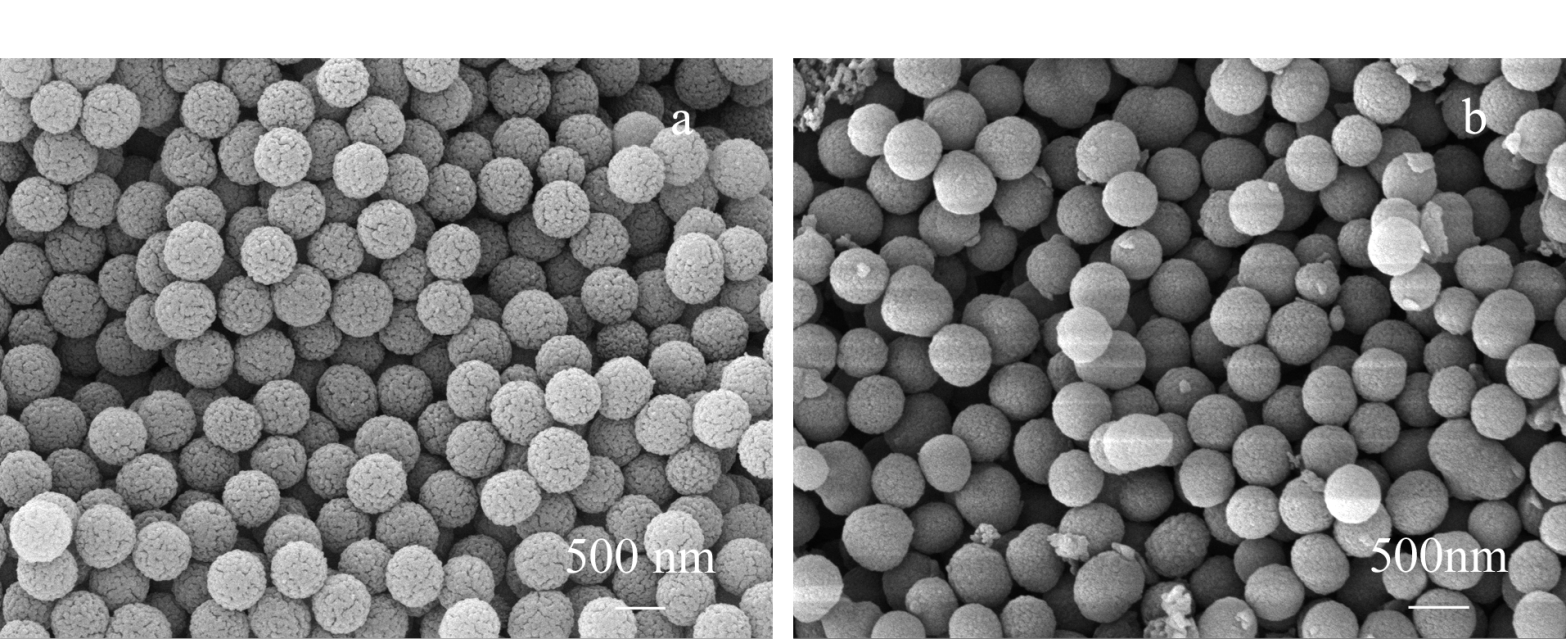
(a)乙烯基COF和(b)氨基COF的SEM表征图

**图4:**
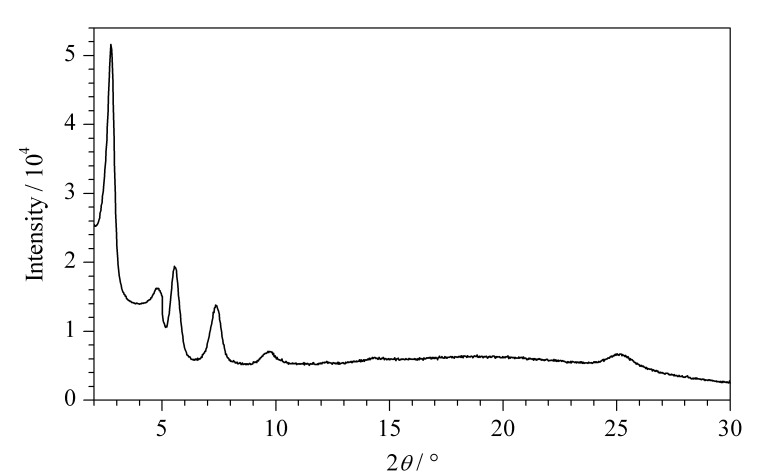
乙烯基COF的PXRD图

**图5:**
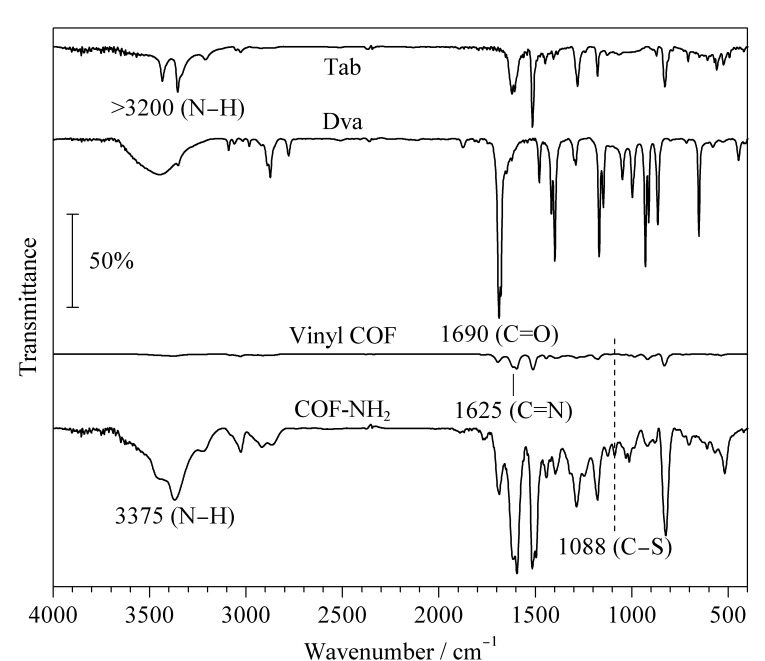
Tab、 Dva、乙烯基COF和氨基COF的红外光谱图

本实验以4-氨基苯硫酚为氨基功能化单体,通过巯醇-烯点击反应将氨基苯修饰在乙烯基COF上,所制备的COF-NH_2_材料依然呈现为单分散性良好的、直径约500 nm的球形,但表面比乙烯基COF稍光滑一些(图3b)。对比乙烯基COF,在COF-NH_2_的红外光谱(图5)中,3375 cm^-1^处N-H特征峰的强度得到了明显提高,而且在1088 cm^-1^处出现了C-S特征峰,这说明已经通过巯基-烯点击反应将氨基成功修饰在乙烯基COF上。

通过N_2_吸附-脱附实验考察了乙烯基COF和COF-NH_2_的比表面积和孔容(图6)。在77 K条件下,两种材料的N_2_吸附-脱附等温曲线相似。在相对低压区氮气的吸附量增加,这是Ⅰ型N_2_吸附-脱附等温曲线的特征,说明材料具有微孔;同时,在脱气过程中出现了一个小的滞后环,这是Ⅳ型N_2_吸附-脱附等温曲线的特征,说明材料存在介孔。乙烯基COF和COF-NH_2_气体吸附类型均为Ⅰ和Ⅳ型的结合,说明两种材料都包含微孔和介孔。乙烯基COF的BET比表面积为224.5 m^2^/g,孔容为0.11 cm^3^/g(*p/p*_0_=0.90)(图6a); COF-NH_2_的BET比表面积为213.4 m^2^/g,孔容为0.10 cm^3^/g(*p/p*_0_=0.90)(图6b)。

**图6:**
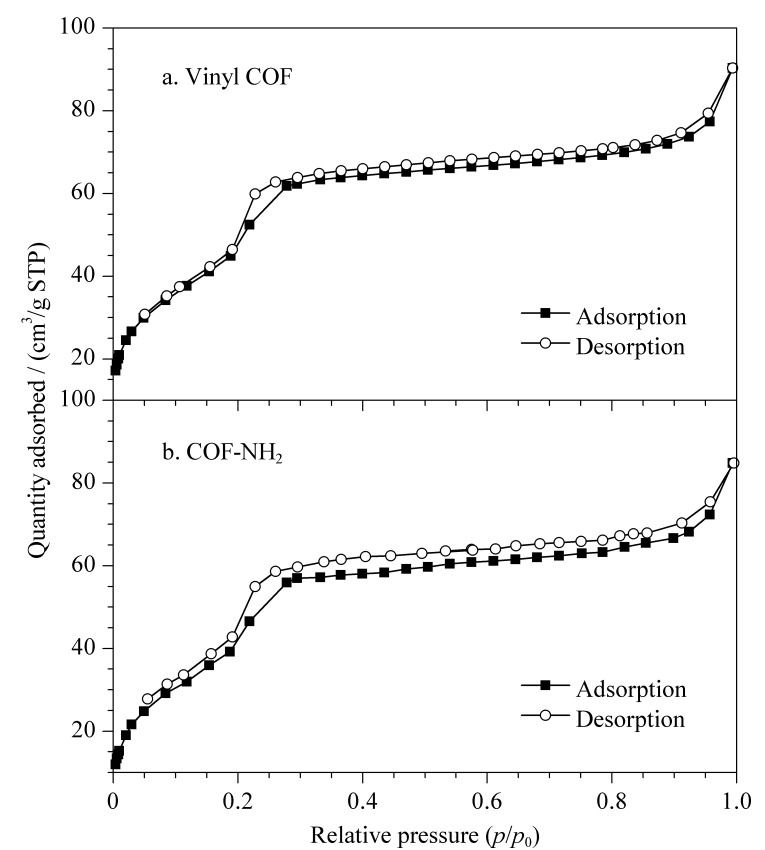
(a)乙烯基COF和(b)氨基COF的N_2_吸附-脱附等温曲线

通过热重法(TGA)考察COF材料的稳定性(图7)。N_2_氛围下,在450 ℃之前乙烯基COF的失重为7.1%,这是骨架孔穴里空气、水分子及有机小分子的排出引起的失重。当温度继续上升至479.5 ℃时,乙烯基COF的失重为10%。当温度上升至800 ℃时,乙烯基COF仅失重31.3%。结果表明,乙烯基COF材料具有很好的稳定性,在高温下其骨架仅部分被破坏。

**图7:**
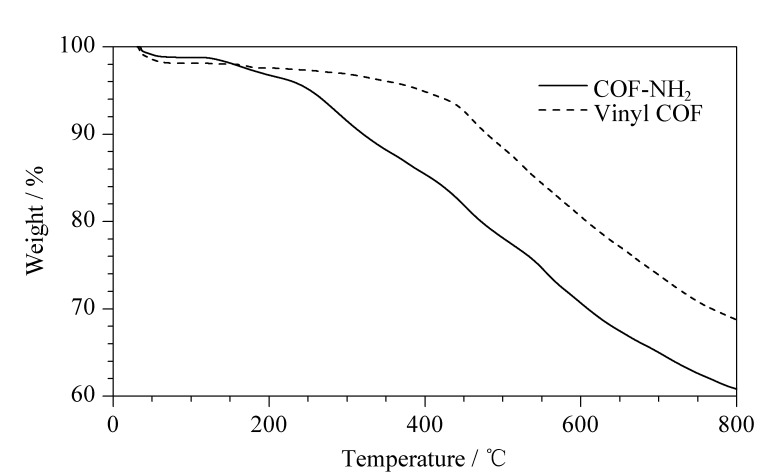
乙烯基COF及氨基COF的TGA表征图

在同样条件下对COF-NH_2_进行TGA分析。N_2_氛围下,在253 ℃之前COF-NH_2_的失重为5%,这同样是共价有机骨架孔穴中的空气、水分子及有机小分子的排出引起的失重。当温度继续上升至800 ℃, COF-NH_2_的失重达到39.8%,这期间的失重可能是由于NH_2_官能团的热解,以及部分COF骨架被破坏。TGA结果表明,乙烯基COF和COF-NH_2_具有足够的稳定性,可应用于实际环境样品的吸附处理。

### 2.2 吸附性能考察

#### 2.2.1 吸附动力学

吸附平衡时间是评价吸附剂吸附性能的重要标准之一。以PFOA为例考察了吸附时间对吸附效率的影响(图8)。由于COF本身具有有序多孔结构及高比表面积的特点,而且COF-NH_2_表面具有氨基官能团,能够通过氢键及静电作用与PFOA结合,COF-NH_2_对于PFOA的吸附可以在6 h内达到平衡并且吸附效率接近100% (图8a)。为了保证PFOA的完全吸附,在随后的实验中,将COF-NH_2_对PFOA的吸附时间控制在6 h。

**图8:**
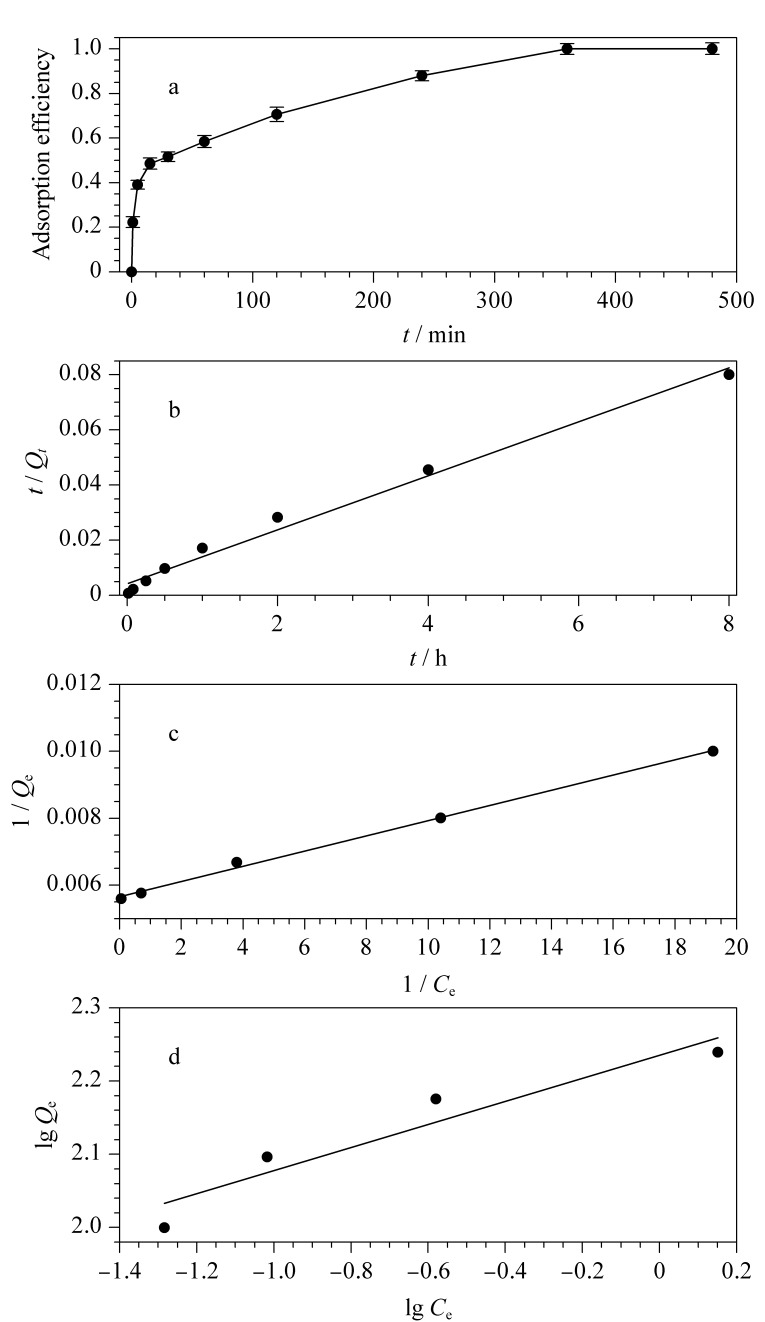
氨基COF对全氟辛酸的吸附及拟合曲线

通过准二级吸附动力学方程(3)分析COF-NH_2_对PFOA的动力学吸附数据:


(3) $\frac{t}{Q_{t}}=\frac{t}{Q_{e}}+\frac{1}{k_{2}Q_{e}^{2}}$

准二级吸附动力学拟合曲线如图8b所示,其中二级动力学常数*k*_2_为0.0229 g/(mg·h),对于100 mg/L的PFOA溶液,该材料的平衡吸附量*Q*_e_为102.04 mg/g,与实验结果相符;线性相关系数(*R*^2^)为0.9876,表明该材料符合准二级动力学吸附,推断其吸附原理主要基于COF-NH_2_材料上的氨基与PFOA分子中F和羧基之间的氢键及静电作用,以及有机骨架与PFOA碳链之间的疏水作用。化学吸附在吸附过程中需克服活化能,故吸附较为缓慢,吸附过程表现在动力学曲线上会出现骤然上升的阶段与缓慢上升的阶段,最终达到吸附平衡(图8a)。

#### 2.2.2 等温吸附模型分析

等温吸附曲线不仅可以用来考察材料的吸附容量,还可用来研究吸附机理。改变PFOA浓度,在298.15 K条件下进行吸附实验。分别通过Langmuir模型(式4)和Freundlich模型(式5)分析COF-NH_2_对PFOA的等温吸附数据。


(4) $\frac{1}{Q_{e}}=\frac{1}{Q_{max}bC_{e}}+\frac{1}{Q_{max}}$

(5) $lg Q_{e}=\frac{lg C_{e}}{n}+lg K_{F}$

式(4)中*Q*_max_代表最大吸附容量,*b* (L/mg)是Langmuir常数。式(5)中*K*_F_ (L/g)表示Freundlich常数,*n*是与吸附亲和力和强度相关的弗兰德里希常数,1/*n*代表分均质系数。如果1/*n*>1,则反映吸附主要是物理过程,反之则反映吸附主要是化学过程。

如图8所示,Langmuir等温拟合曲线(图8c)的线性相关系数为0.9974,而Freundlich模型(图8d)的线性相关系数为0.9085。结果表明COF-NH_2_对PFOA的吸附过程主要是发生在均质表面的单层吸附。通过Langmuir线性方程计算得出COF-NH_2_的最大吸附容量*Q*_max_为175.44 mg/g。通过Freundlich线性方程计算得出*n*为6.37,即1/*n* <1,证实吸附过程主要为化学吸附。

#### 2.2.3 吸附机理

COF-NH_2_的吸附机理示意图如图9所示。COF-NH_2_材料表面具有丰富的氨基,可以与全氟化合物分子中多个氟基或羧基分别形成多重氢键或离子对;此外,COF材料具有多孔性而且含有丰富的苯环,COF骨架与全氟化合物碳链之间的疏水作用可增加协同吸附作用,从而达到较为稳定的吸附效果。

**图9:**
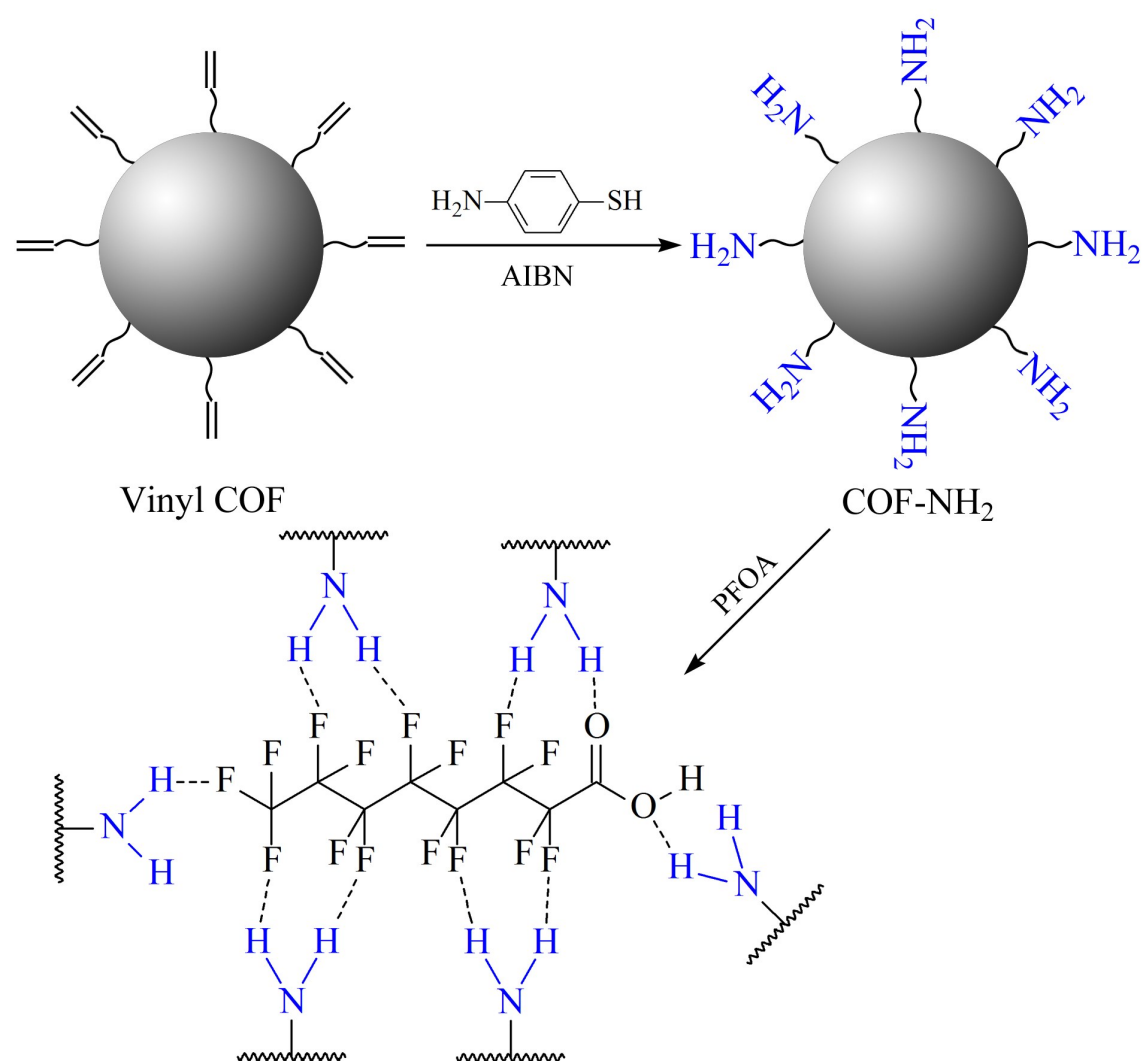
氨基COF的吸附机理示意图

#### 2.2.4 pH的影响

为考察样品溶液酸碱度对COF-NH_2_吸附性能的影响,在pH为4~10的范围内考察了0.5 mg吸附剂对100 mg/L PFOA的吸附效果。如图10所示,在中性偏碱性范围内,COF-NH_2_吸附效果较好;而随着pH降低,COF-NH_2_的吸附效果会逐渐减弱,但吸附效率也都保持在80%以上。这是由于COF-NH_2_主要通过氢键作用与PFOA结合,当pH较低时氨基质子化程度高,氢键作用受到抑制,因而吸附效果变弱。

**图10:**
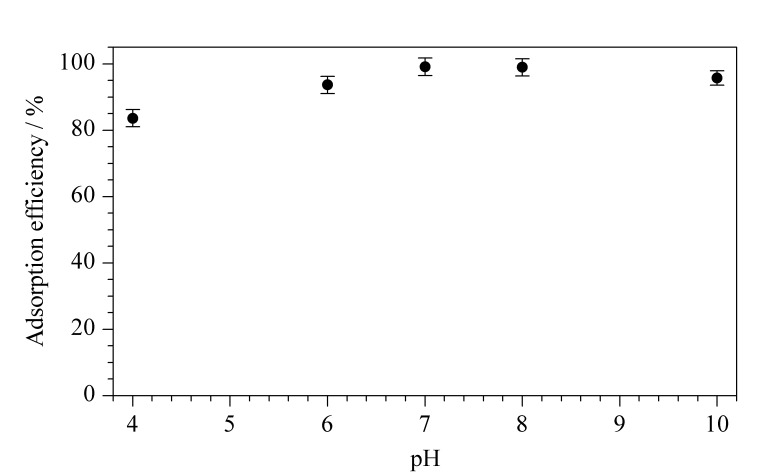
pH对氨基COF吸附PFOA的影响

#### 2.2.5 对不同种类PFCs的吸附分析

在pH=7的条件下考察COF-NH_2_对5种PFCs(PFBA、PFPeA、PFHxA、PFOA和PFNA)的吸附效果。在2 mL离心管中依次加入1.0 mg COF-NH_2_和1 mL 5种全氟化合物(质量浓度均为10 mg/L)的混合溶液,在25 ℃下振荡6 h后,对上清液进行检测。结果发现,上清液中所有PFCs的信号已消失,说明氨基COF材料能够对不同种类的PFCs达到全部吸附的良好效果。如图9所示,该氨基COF材料具有多重作用位点,能够与PFCs碳链上的多个氟基产生氢键;而且,骨架苯环与PFCs碳链之间的疏水作用力可增加协同吸附作用,因此COF-NH_2_对PFCs化合物都能产生较好的吸附效果。

#### 2.2.6 解吸与再生

对COF-NH_2_进行吸附-脱附实验,探究吸附剂的重复使用性。以甲醇为解吸剂,对COF-NH_2_进行5个吸附-脱附循环研究。COF-NH_2_在经过5次吸附-脱附循环实验后,对不同种类PFCs的吸附效率均保持在98%以上,说明该材料具有良好的重复使用性。

### 2.3 氨基COF对实际水体中PFCs的吸附

为了进一步考察氨基COF对实际水体中复杂多样基质的抗干扰能力,以及在实际水体中对PFCs的吸附/去除效果,本实验采集了珠江水及自来水样品进行检测并进行吸附实验,按照1.3节的条件,以HPLC-MS/MS测定水样中PFBA、PFPeA、PFHxA、PFOA和PFNA的含量。结果表明在所采集的自来水和珠江原始水样中均未检出PFBA、PFPeA、PFHxA、PFOA和PFNA,故对加标水样进行吸附研究。分别向1 mL自来水和珠江水样品中加入100.0 mg/L PFBA、PFPeA、PFHxA、PFOA和PFNA,然后将1 mg吸附剂投入到水样中,振荡吸附6 h后,取上清液用0.22 μm微孔滤膜过滤,以HPLC-MS/MS测定PFBA、PFPeA、PFHxA、PFOA和PFNA浓度。结果如表2所示,1 mg COF-NH_2_材料对自来水和珠江水样中5种PFCs的平均吸附效率(*n*=3)分别为94.31%~98.59%和91.76%~98.32%。与自来水相比,珠江水中无机电解质以及有机物都较多,所以珠江水中PFCs的吸附效率稍低于自来水。这说明基质的干扰是存在的,但是基质的影响不大,该材料在复杂水体中具备良好的抗干扰能力和吸附富集效果。

**表2:** COF-NH_2_对实际样品中全氟化合物的吸附效果

Analyte	Tap water			Pearl River water	
	Adsorption efficiency	RSD (n=3)		Adsorption efficiency	RSD (n=3)
PFBA	98.59	1.1		98.32	1.3
PFPeA	97.26	3.5		91.76	2.6
PFHxA	94.31	2.4		93.52	3.8
PFOA	98.19	0.82		97.95	0.94
PFNA	95.82	2.1		92.64	3.2

## 3 结论

本文基于功能化COF策略,首先室温合成出球形乙烯基COF材料,然后通过点击反应对其修饰,将氨基修饰于COF上合成出单分散性良好,并具有良好热稳定性的球形COF-NH_2_。该材料可以利用骨架上的氨基与PFCs分子中多个氟基或端基羧基分别形成多重氢键或离子对,并通过骨架苯环与PFCs碳链之间的疏水协同作用力来有效吸附多种PFCs。重复再生5次后,吸附效果几乎无影响。该材料具备应对复杂水体的抗干扰能力,对自来水和珠江水样品中的5种PFCs (PFBA、PFPeA、PFHxA、PFOA和PFNA)的吸附效率介于91.76%与98.59%之间。该球形COF材料尺寸均匀、热稳定性好,具有较好的吸附性能而且容易再生,具有较好的应用前景,可望用作SPE材料或者填充到液相色谱柱中用于PFCs的高效富集分离。另外,研究表明,有机化合物受到抗磁力推动,会趋向于运动到磁场最弱的地方^[27]^。如果在萃取阶段施加与样品流动方向相同的磁场,可增加PFCs在SPE柱上的富集效率;然后在洗脱阶段,施加与溶剂流动方向相反的磁场,则可推动PFCs与解吸液一同流出来,提高洗脱效率。因此将COF-NH_2_微球进一步修饰,制备出磁性微球,建立磁场增强-管内固相萃取方法,可望进一步提高样品中PFCs的萃取富集效率,目前课题组正在进行此项研究。
